# Identification and characterization of the cupin_1 domain-containing proteins in ma bamboo (*Dendrocalamus latiflorus*) and their potential role in rhizome sprouting

**DOI:** 10.3389/fpls.2023.1260856

**Published:** 2023-10-16

**Authors:** Peng-kai Zhu, Jing Yang, De-ming Yang, Yan-ping Xu, Tian-you He, Jun-dong Rong, Yu-shan Zheng, Ling-yan Chen

**Affiliations:** ^1^ College of Landscape Architecture and Art, Fujian Agriculture and Forestry University, Fuzhou, China; ^2^ College of Forestry, Fujian Agriculture and Forestry University, Fuzhou, China

**Keywords:** cupin_1 domain, rhizome sprouting, *Dendrocalamus latiflorus*, bamboo shoot development, co-expression

## Abstract

Cupin_1 domain-containing protein (CDP) family, which is a member of the cupin superfamily with the most diverse functions in plants, has been found to be involved in hormone pathways that are closely related to rhizome sprouting (RS), a vital form of asexual reproduction in plants. Ma bamboo is a typical clumping bamboo, which mainly reproduces by RS. In this study, we identified and characterized 53 *Dendrocalamus latiflorus* CDP genes and divided them into seven subfamilies. Comparing the genetic structures among subfamilies showed a relatively conserved gene structure within each subfamily, and the number of cupin_1 domains affected the conservation among *D. latiflorus* CDP genes. Gene collinearity results showed that segmental duplication and tandem duplication both contributed to the expansion of *D. latiflorus* CDP genes, and lineage-specific gene duplication was an important factor influencing the evolution of CDP genes. Expression patterns showed that CDP genes generally had higher expression levels in germinating underground buds, indicating that they might play important roles in promoting shoot sprouting. Transcription factor binding site prediction and co-expression network analysis indicated that *D. latiflorus* CDPs were regulated by a large number of transcription factors, and collectively participated in rhizome buds and shoot development. This study significantly provided new insights into the evolutionary patterns and molecular functions of CDP genes, and laid a foundation for further studying the regulatory mechanisms of plant rhizome sprouting.

## 1 Introduction

Organ growth regulation plays an important role in the future challenges that face us to meet the increasing demand for food, feed, and biofuels (Nelissen and Gonzalez, 2019). The identification and characterization of genes that are regulated for plant growth and how they are coordinated for development is crucial in plant biology. The cupin superfamily has a wide range of functions in multiple processes, such as seed storage, growth and development, stress resistance and metabolic regulation (Khuri et al., 2001; Brunetti et al., 2022; Peng et al., 2022).

The cupin_1 domain-containing protein (CDP) family is based on the Pfam domain cupin_1 (PF00190). It belongs to the cupin superfamily, which harbors one or more cupin_1 domains (Y. He et al., 2022). This domain is a conserved structural domain with the capacity to bind metal ions, which contains two conserved motifs of β-strands separated by a less conserved region consisting of another two β-strands with a variable loop in between (Rocha et al., 2021). The CDP family can be classified into monocupin, bicupin and multicupin groups, which have one, two or more than two cupin_1 domains, respectively (Dunwell et al., 2004). Monocupin comprises some important plant proteins, such as 11S and 7S plant seed storage proteins, germin, germin-like protein (GLP) and so forth (Dunwell et al., 2004; Stipanuk et al., 2011). The GLP proteins are derived from the tissue-specific posttranslational processing of the proglucagon peptide. For example, GLP proteins in *Cucumis melo* responded to low temperatures, and *CmGLP2-5* may have a key role in this process (Zhang et al., 2022). Overexpression of *DIGLP1-5-1* in the globular embryos of *Dimocarpus longan* increased the accumulation of lignin and lowered the H_2_O_2_ content by modulating the activities of ROS-related enzymes (Li et al., 2023).

Nevertheless, most studies have not simultaneously identified and characterized bicupins or muticupins that contain two or more cupin_1 domains. Recently, all members of the CDP family in *Brassica napus* were identified and characterized, and two BnCDPs were found to commonly respond to multiple stresses (He et al., 2022). This laid a foundation for a comprehensive understanding of the functions of the CDP family and set a precedent for further studies on this topic.

Rhizome sprouting (RS) refers to the capacity of plants to propagate and proliferate through the germination of rhizome buds. This intricate process of shoot emergence is heavily governed by the involvement of plant hormones (Bessho-Uehara et al., 2018; Wang et al., 2019). Previous research has unveiled the engagement of CDP genes in pathways related to auxin metabolism (Khuri et al., 2001; Strader and Zhao, 2016). For example, one of the CDP members is Auxin-binding protein 1 (*ABP1*), which binds the plant hormone auxin, indole-3-acetic acid (Napier, 2021). There is evidence that ABP1 has some roles in auxin-mediated responses (Tromas et al., 2009). Furthermore, *GLP4*, a Golgi-localized member of the CDP family, has been demonstrated to bind auxin *in vitro* (Yin et al., 2009). This collective evidence underscores the involvement of CDP genes in plausible auxin-related pathways. Yet, within our current purview, the role of CDPs in RS remains enigmatic.

Ma bamboo (*Dendrocalamus latiflorus*), stands as a pivotal giant bamboo species within the South Asian region (Xiang et al., 2021). It assumes a noteworthy role by bestowing local economies with a repertoire of valuable resources, namely consumable shoots and timber provisions (Ye et al., 2017; Wang et al., 2021). Notably, categorized as a RS plant, bamboo exhibits an emblematic life cycle (Shou et al., 2020). Bamboo shoots materialize through the development of rhizome buds, which engage in a rhizome-shoot system that thereby orchestrating the expansion of its population (Jang et al., 2006; Wang and Li, 2008; Zheng et al., 2022).

Prior investigations have underscored the multifaceted influences regulating the germination rate of rhizome buds (McIntyre, 1976; Leakey et al., 1978; McIntyre, 1987; Wang et al., 2010). The varying factors affecting ma bamboo shoot yield in commercial cultivation have led to fluctuations. To address this, we suggest exploring certain genes as an alternative avenue. This could illuminate the genetic mechanisms controlling bamboo shoot germination, potentially reducing shoot emergence unpredictability. However, understanding genetic mechanism of rhizome sprouting in Bambusoideae, including Ma bamboo, remains limited, leaving a noticeable gap in knowledge.

The present study initiates with the identification and characterization of CDPs in Ma bamboo. Subsequently, by scrutinizing differentially expressed genes (DEGs) within germinating and dormant rhizome buds, we extrapolate the potential molecular functions of CDPs. Moreover, we elucidate a co-expression network between CDPs and transcription factors (TFs) during the rhizome bud-to-shoot transition. These endeavors collectively lay a foundation for further elucidating the molecular mechanisms of bamboo rhizome bud development and provide new insights into the broader biological functions of CDPs.

## 2 Method

### 2.1 Plant materials and transcriptome data analysis

Bamboo Botanical Garden of Fujian Agriculture and Forestry University is located in Cangshan District, Fuzhou City, Fujian Province, China (N26°05’, E119°14’). To understand the process of *D. latiflorus* shoot development, we collected germinative rhizome buds and dormant rhizome buds at the same location, which are newly generated data in this study. To reduce experimental errors, three biological replicates were collected for each sample. The newly generated transcriptome data were aligned to the reference genome using Hisat2 (Kim et al., 2015) and quantified using Htseq 2.0 (Putri et al., 2022), and differentially expressed genes were screened using Deseq2 (|log_2_ (Fold Change) |≥1 & Padj ≤ 0.05) (Love et al., 2014). Then the transcripts per kilobase of exon model per Million mapped reads (TPM) value of DlCDP genes (DlCDPs) were extracted from all transcriptome data. After normalizing log_2_(TPM+1), a heat map was generated using TBtools (Chen et al., 2020). Correlation analysis of DlCDPs and TFs based on Pearson Correlation Coefficients (PCCs) was performed using Python script. Cytoscape 3.10.0 (Shannon et al., 2003) was used to visualize the co-expression network. GO enrichment analysis was performed using Clusterprofiler software (Wu et al., 2021).

### 2.2 q-PCR validation

Total RNA was isolated from germination buds and dormant buds using RNA prep Pure Plant Kit (Tiangen, Beijing, China), and reverse transcribed into cDNA using PrimeScript™ RT reagent Kit (Perfect Real Time, Takara, Japan). Quantitative real-time PCR analysis of 8 selected DlCDPs was performed on a LightCycler480 instrument (Roche) using Taq Pro Universal SYBR qPCR Master Mix SYBR Green premix Ex Taq Kit (TaKaRa, Dalian, China) on an Applied Biosystems 7500 Real-Time System (Applied Biosystems, Foster City, CA, USA). The q-PCR amplification program was: 95°C pre-denaturation for 5 seconds, 95°C denaturation for 30 seconds, 60°C annealing for 30 seconds; 72°C extension for 30 seconds, 40 cycles. GAPDH gene was used as an internal reference gene (Liu et al., 2014), and the relative expression of each gene was calculated using the 2-ΔΔCT method. The primers used in this study are shown in **Table S5**.

### 2.3 Identification of *D. latiflorus* CDP gene family

The genome and annotation information of *D. latiflorus* was obtained from DPGD (http://forestry.fafu.edu.cn/pub/Dla/index.html). We adopted a workflow similar to previous studies (Lozano et al., 2015), utilizing the HMM profile PF00190 from Pfam (Mistry et al., 2021). Initially, we applied the raw cupin_1 HMM to search high-quality proteins with an e-value of 1e^-20^. Then, we build species-specific HMM models and search all proteins using an e-value threshold of 0.01 to identify cupin_1 proteins in the *D. latiflorus* genome. Subsequently, the integrity of the putative CDP genes was further examined by InterPro (http://www.ebi.ac.uk/interpro/search/sequence/) (Paysan-Lafosse et al., 2023).

### 2.4 Bioinformatic analysis of *D. latiflorus* CPD gene family

The physicochemical properties, transmembrane domains, signal peptides and subcellular localization of DlCDP members were analyzed using ExPASy (https://web.expasy.org/protparam/) (Gasteiger, 2003), DeepTMHMM (https://dtu.biolib.com/DeepTMHMM/) (Hallgren et al., 2022), SignalP 6.0 (https://services.healthtech.dtu.dk/services/SignalP-6.0/) (Teufel et al., 2022) and WoLF PSORT (https://wolfpsort.hgc.jp/) (Horton et al., 2007) respectively. We used MEME (https://meme-suite.org/meme/doc/meme.html) (Bailey et al., 2006) to identify the conserved motifs of DlCDPs, with the parameters set as follows: maximum e-value=1e-5, minimum motif length=6, maximum motif length=200, and number of motifs=15. Subsequently, we produced conserved domain sequence logos and a gene structure view of DlCDPs using TBtools. We extracted the 2000 bp sequences upstream of the start codon (ATG) of each DlCDP gene, and predicted the cis-elements and transcription factor binding sites (TFBS) using PlantCARE (http://bioinformatics.psb.ugent.be/webtools/plantcare/html/) (Lescot, 2002) and PlantTFDB (http://plantregmap.gao-lab.org/index.php) (Jin et al., 2017), respectively. All results were then visualized using TBtools.We performed protein-protein interaction analysis on the STRING database (http://string-db.org), using DlCDPs as queries, and built a protein interaction network based on their homologs in *Arabidopsis thaliana*.

### 2.5 Phylogeny, gene duplications and selection pressure analysis

To investigate the phylogenetic relationship of CPDs from *D. latiflorus*, we searched for homologs of DlCDPs in the model plants *Arabidopsis thaliana*, *Oryza sativa* and *Miscanthus sinensis* using Blastp in the Pythome (https://phytozome-next.jgi.doe.gov/) (Goodstein et al., 2012) with a maximum e-value of 1e-20. We then aligned these genes using Muscle 3.8 (Edgar, 2004), and trimmed the alignment result automatically using trimAL 1.4 (Capella-Gutiérrez et al., 2009). We constructed a maximum likelihood (ML) tree using FastTree 2.1.10 (Price et al., 2010) with default parameters, and visualized the phylogenetic tree using iTOL (Letunic and Bork, 2021). To better illustrate the phylogenic relationship among DlCDPs, we manually cut some branches without DlCDPs.

We obtained the subgenome assignment, chromosomal position and exon number of DlCDP genes from the annotation file of the *D. latiflorus* genome. We used Blast to compare the similarity of all *D. latiflorus* protein sequences, and MSCanX (Wang et al., 2012) to generate information on fragments and tandem repeats. We visualized the collinearity among DlCDPs using the Advanced Circos module in TBtools. We also analyzed the orthologs of DlCDPs across species in the same way, and visualized the results using NGenomeSyn 1.41 (He et al., 2023). We estimated the non-synonymous substitution (Ka) and synonymous substitution (Ks) values of duplicated DlCDP gene pairs using TBtools.

## 3 Results

### 3.1 CDP genes in *D. latiflorus*

In this study, we identified 14, 17, and 22 new CDP members in the A, B, and C genomes of *D. latiflorus*, respectively (**Table S1**). We renamed them according to their subgenome distribution and chromosomal location. The proteins encoded by these genes ranged in size from 147 to 562 amino acids, and the theoretical molecular weight of DlCDPs varied from 15,057.18 to 63,181 Da, with isoelectric points (pI) ranging from 4.7 to 11.35. Most DlCDP proteins were characterized as unstable and hydrophilic proteins. Signal peptide prediction analysis revealed that 76 DlCDP members contained signal peptide sequences. Transmembrane domain (TM) prediction showed that only *DlCDP44* contained three TMs, while the other members did not have transmembrane structures. Subcellular localization prediction indicated that 26, 16, 6, and 3 DlCDPs were located in the extracellular, chloroplast, vacuole and plastid compartments, respectively, while one member each was located in the mitochondrion and nucleus.

### 3.2 Phylogenetics of the DlCDPs

**Figure 1:**
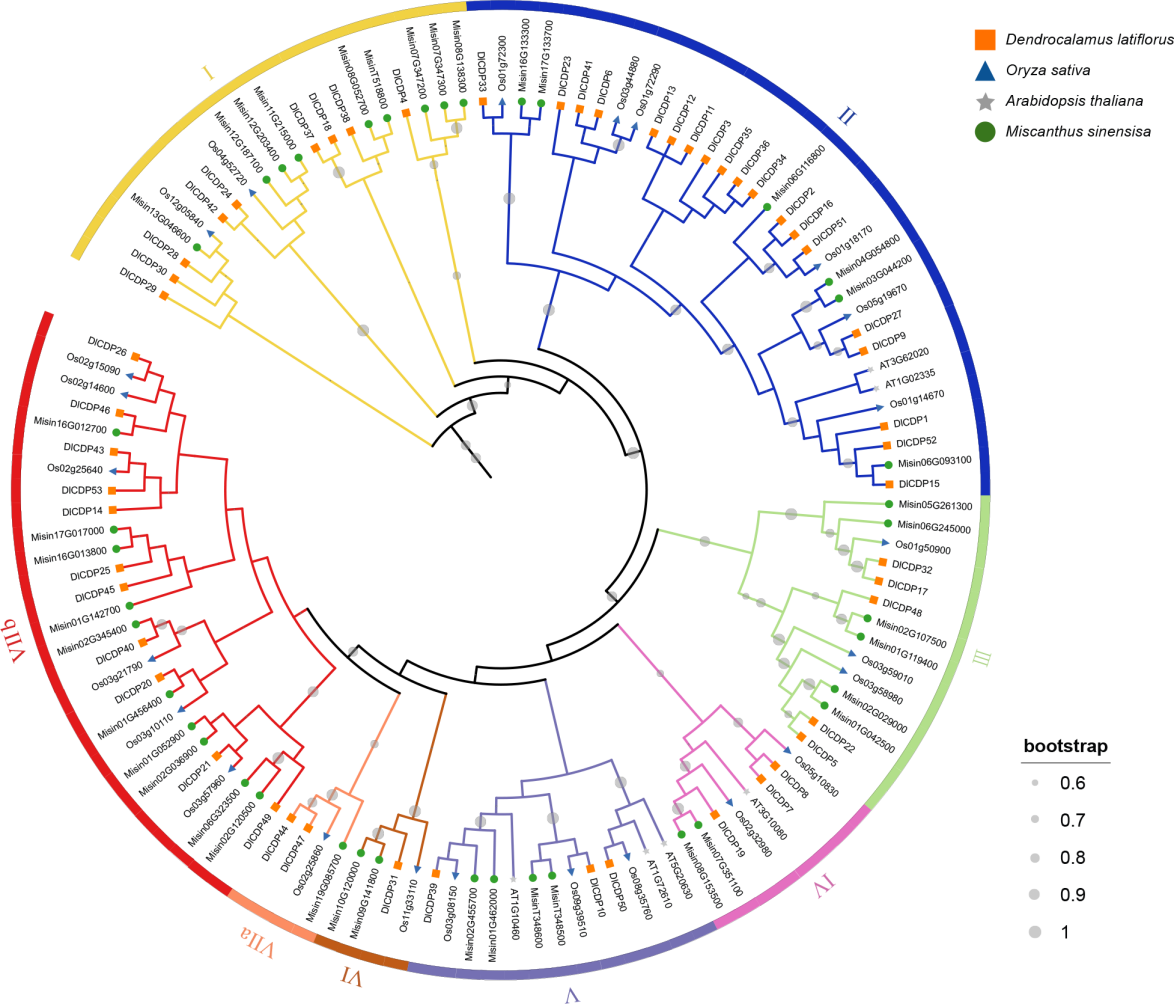
Maximum likelihood (ML) phylogenetic tree of CDP proteins from *D. latiflorus*, *A. thaliana*, *O. sativa* and *M. sinensis*.

To validate the accuracy of our identification method and investigate the evolutionary relationships of CDP genes, we constructed a ML tree using a total of 123 CDPs (**Figure 1**). The results showed that the number of orthologs of DlCDP in *Miscanthus sinensis*, *Oryza sativa*, and *Arabidopsis thaliana* was 40, 24, and 6, respectively. Among the three members of the Poaceae, their CDPs show high homology. Based on the branch length and the number of cupin_1 domains, we classified these CDPs into seven subfamilies (I ~ VII), and further subdivided subfamily VII into two subclades (VII a and VII b). Subfamily I contained almost equal DlCDPs and CDPs from other species, with 9 and 11, respectively. Subfamily II contained the most DlCDPs, with 19, and the fewest CDPs from other species. This suggests that some DlCDPs in subfamily II may have emerged after the divergence of *D. latiflorus* from *O. sativa* and *M. sinensis*, resulting in their low homology with other CDPs. Subfamily III to subfamily VI had fewer DlCDPs, with 5, 3, 3, and 1, respectively.

Moreover, subfamily VII a and VII b had 2 and 11 DlCDPs, respectively, and all bicupins came from subfamily VII b, indicating that bicupins have sequence differences from most monocupins. As we expected, there were many homologous genes in *O. sativa* and *M. sinensis* for the bicupin members of DlCDP, they may play roles different to monocupins. Interestingly, all members of subfamily VII except *DlCDP14* came from subgenome B and C almost exclusively, and all bicupins came from subgenomes B and C as well. This suggests that most bicupins of DlCDPs may have arisen during polyploidization.

### 3.3 Gene structure and conserved protein motifs of DlCDP family

**Figure 2:**
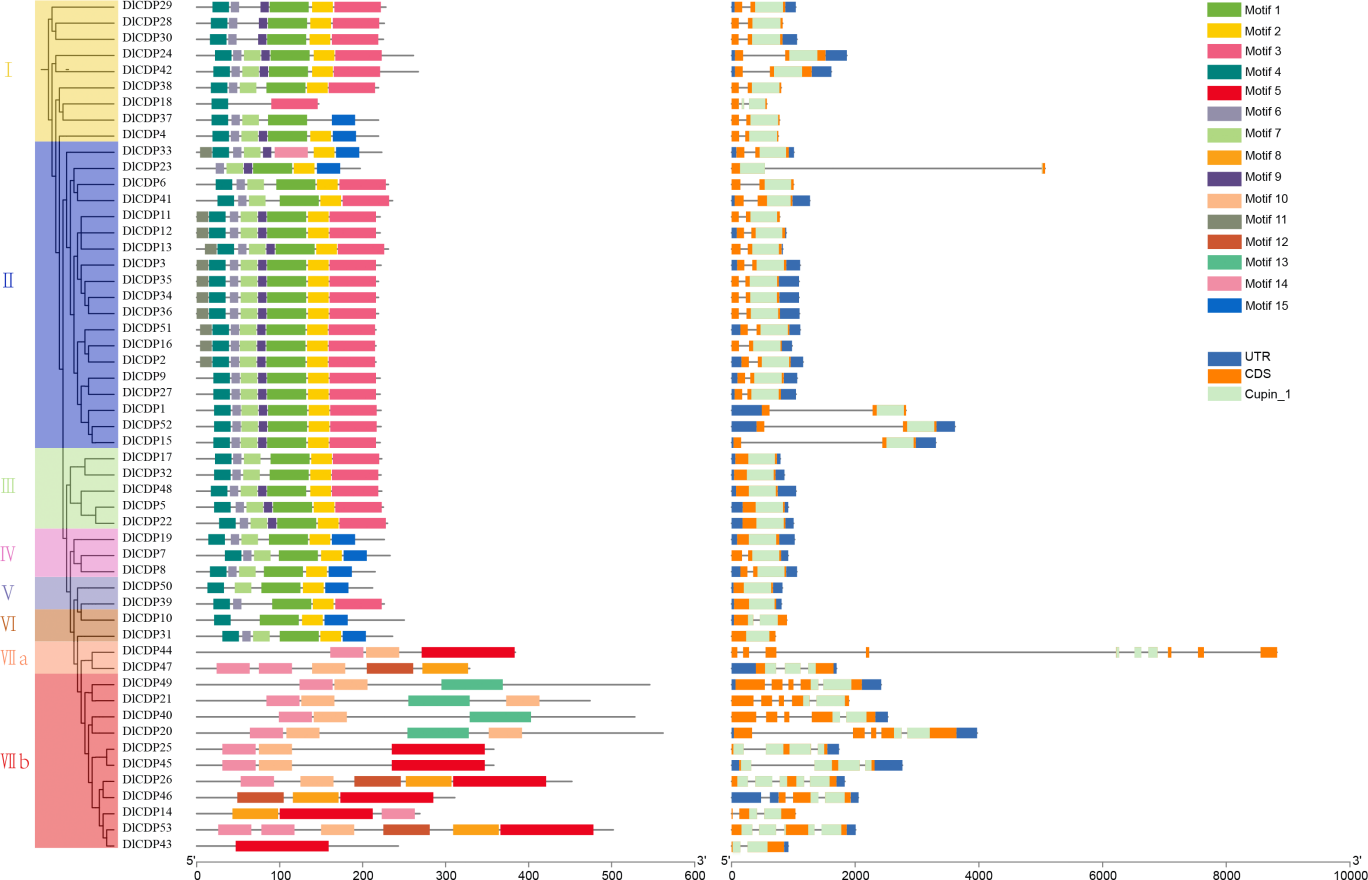
The phylogenetic relationship, conserved motifs, and gene structure diagram of 53 DlCDPs in *D. latiflorus*. Scale bars represent gene length (bp) and protein sequence length (aa).

Using MEME, we detected 15 conserved motifs in DlCDPs and mapped them to pfam domains (**Figure 2**). We also found that motifs 1-3, 5, 8, 10, 13, and 14 matched protein sequences with the cupin_1 domain (**Table S2**). CDP proteins within the same subfamily shared more similar types, numbers, and distributions of conserved motifs. Motif 4, 6, 7 and motif 1-3 are arranged in the same order in most members of subfamily I~VI. However, motif 1 is absent in *DlCDP18*, while motif 2 is absent in *DlCDP18* and *DlCDP37*. DlCDPs of subfamilies IV-VI and I-III share a similar motif order and number, if motif 3 is replaced by motif 15 for all members except *DlCDP39*. Additionally, DlCDPs of subfamilies VIIa and VIIb have motif 14, but not *DlCDP43* and *DlCDP36*. While, motif 5 is the only motif in *DlCDP43*. Furthermore, Motif 10 was absent from *DlCDP46* and *DlCDP10* but present in the rest of the members. In summary, subfamilies I-VI and subfamily VII showed high sequence conservation within each group but low conservation between groups.

We further investigated the structures of DlCDP genes (**Figure 2**), which varied in length from 572 bp (*DlCDP18*) to 8821 bp (*DlCDP44*). *DlCDP44* has the highest number of exons and introns among all members, with ten and nine respectively. Most of the DlCDPs have only two exons and one intron, totaling 31 genes. Moreover, nine DlCDP members (*DlCDP5*, *17*, *19*, *22*, *31*, *32*, *39*, *48*, and *50*) have only one exon and no introns. Most DlCDPs have short exons and introns. However, the longest intron length of the two longest *DlCDPs*, *DlCDP23* and *44*, both exceed 3900 bp. Thus, intron length is the main factor resulting in increase in gene length.

### 3.4 Chromosomal location and gene duplication of DlCDPs

We discovered that the DlCDPs are equally distributed on seven chromosomes in each subgenome. However, the numbers of DlCDPs are unevenly distributed in each chromosome. Chr3.1 and chr15.1, both belonging to subgenome C, carried the most DlCDPs, with five DlCDPs each. The second most abundant were chr25.1 and 32.1, with four DlCDPs each. Six chromosomes each carried two or three DlCDP members. The number of DlCDPs on chr10.1, chr26.1, chr33.1, chr4.1, and chr7.1 was the lowest, with one each.

**Figure 3:**
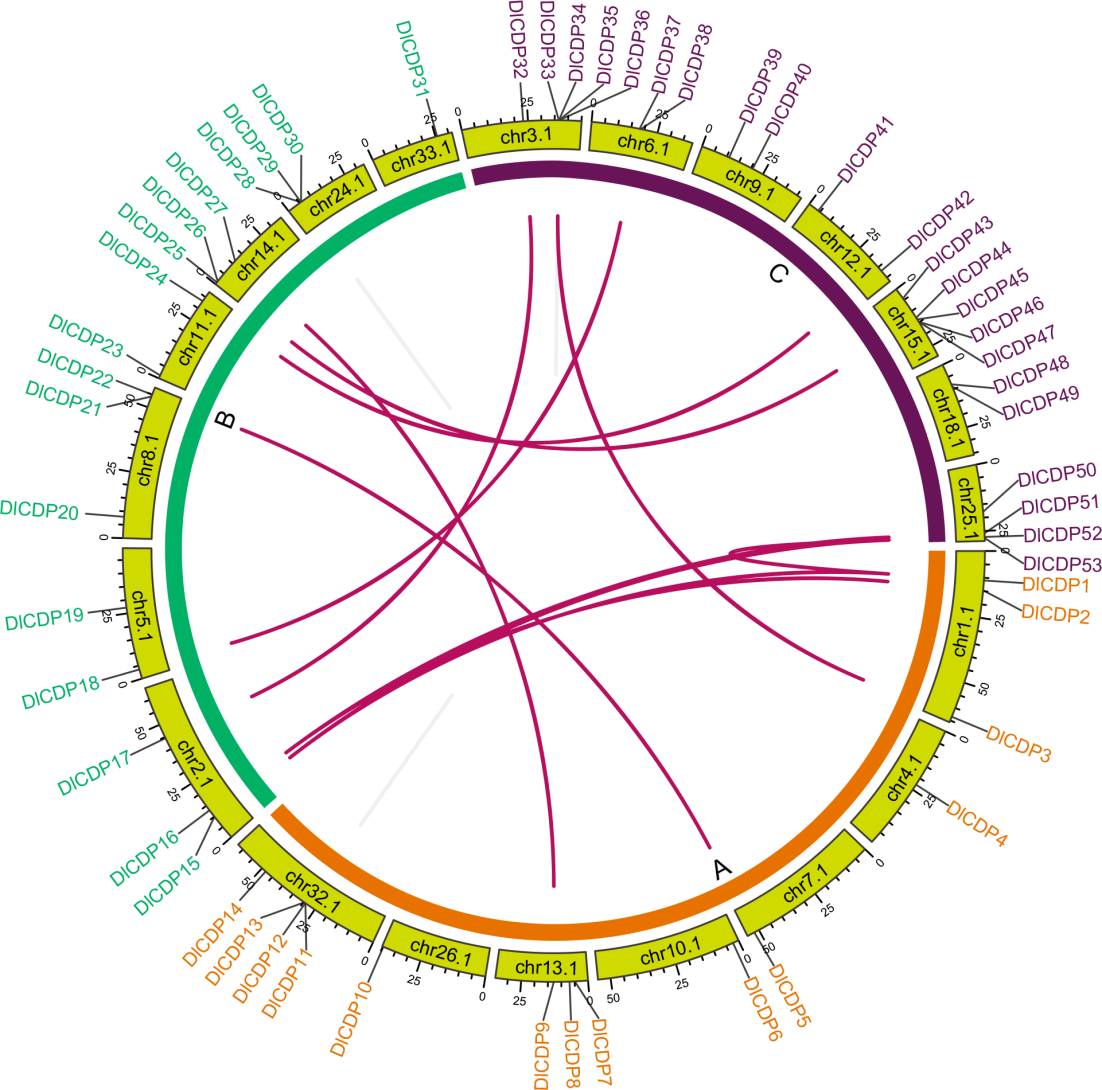
Chromosomal location and collinearity analysis of DlCDP family genes. Chromosomes are represented by light green boxes. Segmental duplication genes are connected with dark red lines. Tandem duplication genes are connected with grey lines.

**Figure 4:**
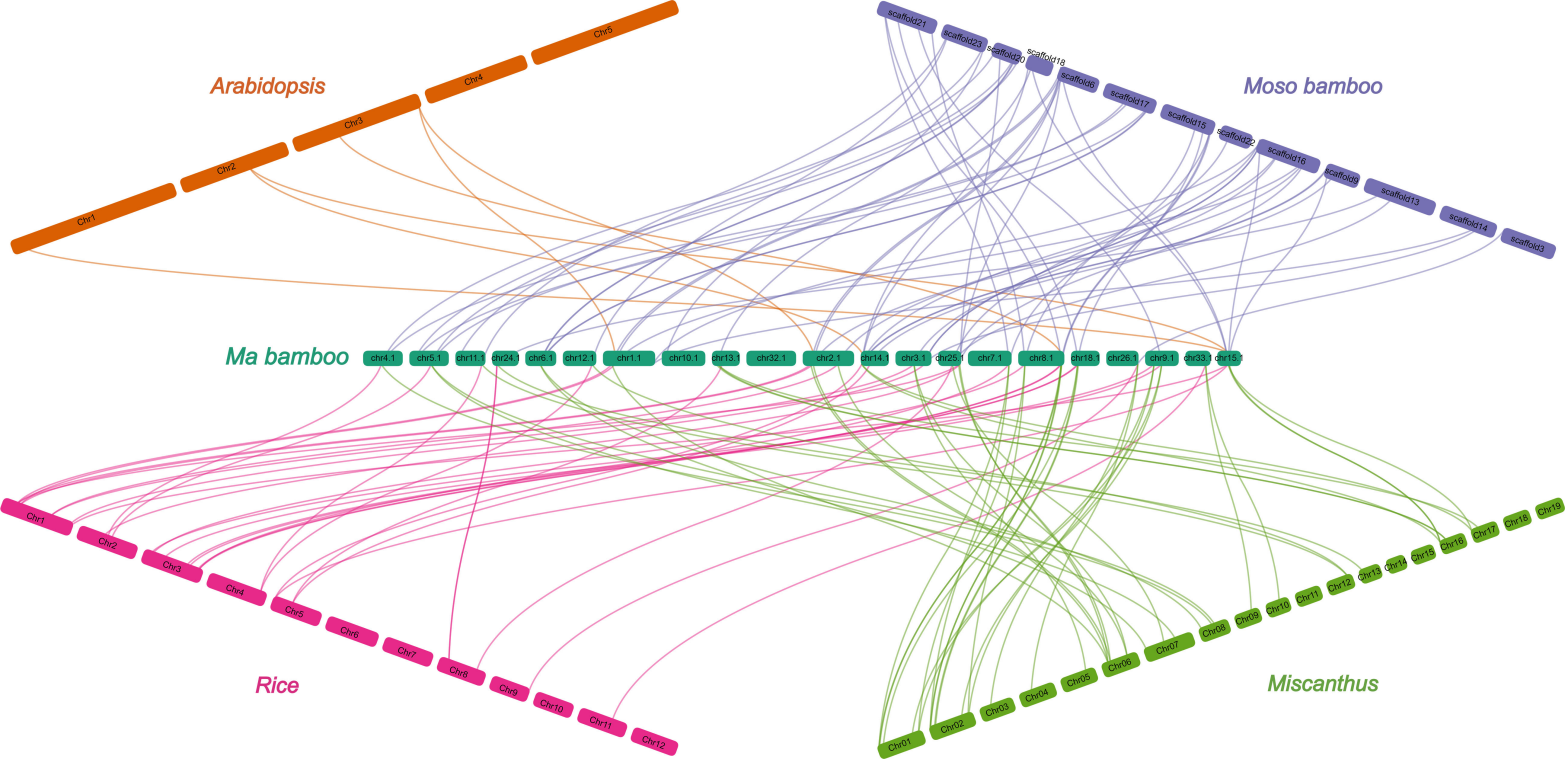
Collinear correlation analysis among five species.

To investigate the gene duplication events within the DlCDP family, we performed homology analysis. We identified 12 pairs of segmental duplication genes and five pairs of tandem duplication genes (**Figure 3**; **Table S3**). All segmental duplication genes occurred between subgenomes. Notably, we found a special segmental duplication event involving three members of the DlCDP family (*DlCDP1*/*DlCDP15*/*DlCDP52*) with similar gene lengths and equal numbers of exons and introns. In addition, Duplicate gene pairs were only found in four subfamilies. subfamily II had the most duplicate gene pairs (10 pairs, including seven segmental and three tandem duplication pairs), followed by subfamily I (four pairs, including two segmental and two tandem duplication pairs). Subfamily III and subfamily VII b had two and one duplicate gene pairs respectively, and both were segmental duplications (**Table S3**). Moreover, we identified homologous genes of DlCDPs in four other species (**Figure 4**). The results showed that DlCDPs formed the most collinear gene pairs with genes from moso bamboo (*Phyllostachys edulis*), with 55 pairs, followed by 41 and 33 collinear gene pairs with *M. sinensis* and *O. sativa* respectively, while only six collinear gene pairs were found with *A. thaliana*. Furthermore, we counted the number of CDP homologous genes in subgenomes A, B and C respectively, and found that in four species 71.43%, 94.12% and 72.73% of genes in subgenomes A, B and C respectively could find homologous genes (**Table 1**).

**Table 1:** Total DlCDPs that are homologous to genes from four species in subgenomes.

D. latiflorus subgenomes	A1		B1		C1	
	No. of DlCDPs	Frequency	No. of DlCDPs	Frequency	No. of DlCDPs	Frequency
*A. thaliana*	1	7.14%	3	17.65%	2	9.09%
*P. edulis*	8	57.14%	14	82.35%	14	63.64%
*O. sativa*	6	42.86%	11	64.71%	12	54.55%
*M. sinensis*	6	42.86%	13	76.47%	11	50.00%
In total	10	71.43%	16	94.12%	16	72.73%

To estimate the functional selection pressure of duplicate gene pairs, we calculated Ka and Ks for DlCDPs and their homologs in four species from Poaceae, where Ka and Ks represent the rates of nonsynonymous and synonymous substitutions per site, respectively (**Table S4**). In general, Ka/Ks>1 indicates that genes are under positive selection; Ka/Ks=1 indicates that genes undergo neutral selection; while Ka/Ks<1 indicates that genes undergo purifying selection. Only four DlCDPs have Ka/Ks ratios greater than 1 compared to the CDPs from the other four species, indicating that they were under positive selection. Whereas, all other DlCDPs have Ka/Ks ratios below 1, and most of the DlCDPs have Ka/Ks ratios below 0.5, reflecting their strong purifying selection and high conservation during evolution.

### 3.5 Cis-acting elements in DlCDP promoters

**Figure 5:**
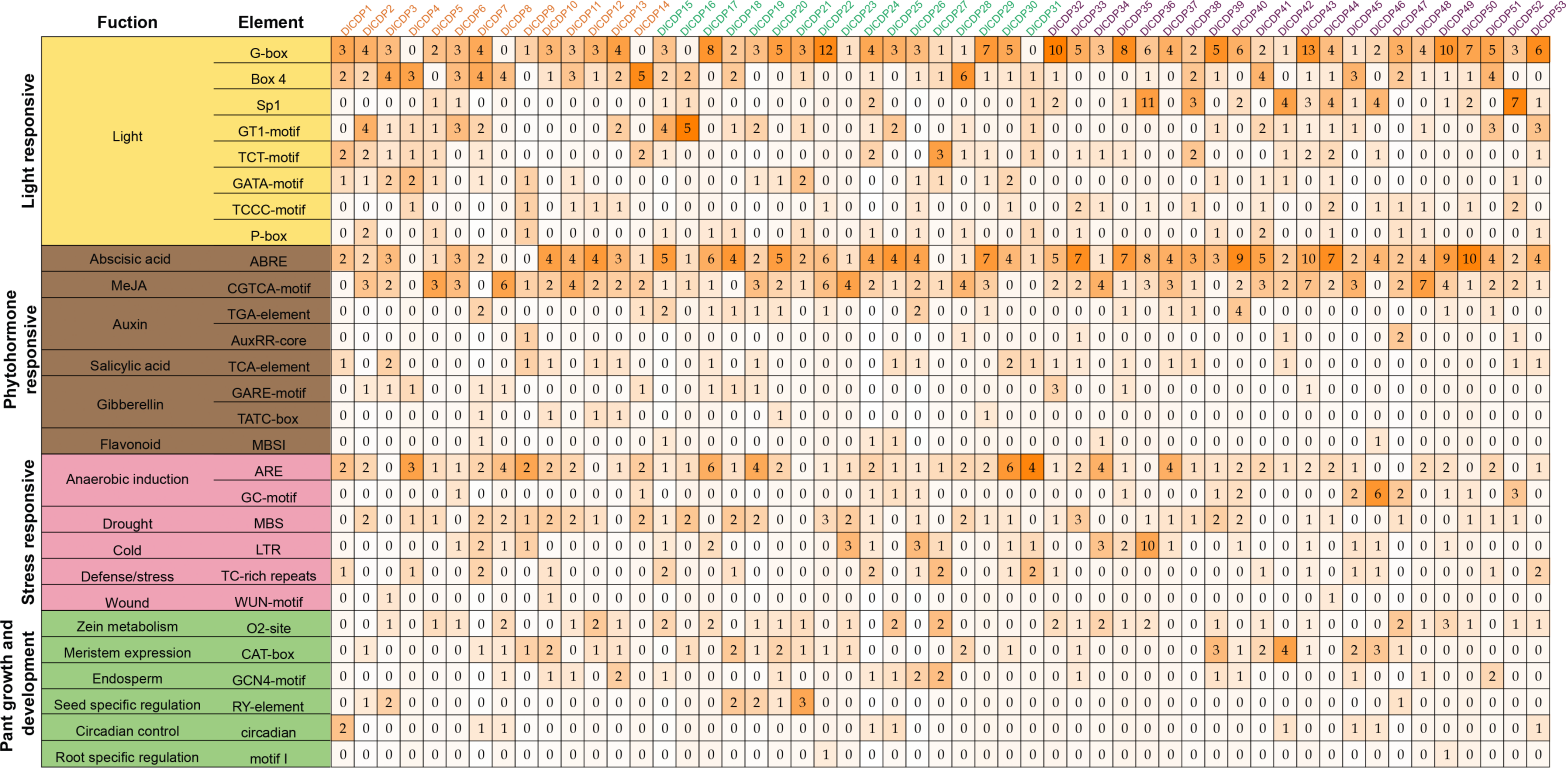
Cis-acting elements in promoters of DlCDP genes.

The promoter is the regulatory region of the gene that contains various cis-acting elements that can respond to environmental stress and regulate gene expression. The cis-acting elements identified in the promoters of DlCDPs could be classified into four categories: light, hormone and stress responsive elements, and elements related to plant growth and development (**Figure 5**). All promoters of DlCDPs contained light responsive elements, with G-box being the most abundant (204) and present in 48 DlCDPs. Abscisic acid (ABA) responsive elements were the most abundant hormone responsive elements (198), and they occurred in all but four DlCDP promoters. The second most common were methyl jasmonate (MeJA) responsive elements (116). Cis-acting elements involved in auxin response (TGA-element and AuxRR-core) were detected in 29 promoters of DlCDPs. Salicylic acid (SA), gibberellin (GA), and flavonoid responsive elements were present in 12, 6, and 6 promoters of DlCDP members respectively. Moreover, all promoters of DlCDPs except *DlCDP39* had at least one type of hormone responsive element, with a maximum of four types. Among them, *DlCDP4* only had one GA responsive element, while *DlCDP43* had the most hormone responsive elements (18).

DlCDP promoter regions contained a variety of stress response elements. Cis-acting elements for anaerobic induction (ARE and GC-motif), drought (MBS), cold (LTR), defense/stress (TC-rich repeats), wound (WUN-motif) were present in 45, 34, 21, 14, 18, and 3 promoters of DlCDPs respectively. Furthermore, some minor but equally important elements related to growth and development were identified. Zein metabolism (O2-site), meristem expression (CAT-box), endosperm (GCN4_motif), seed specific regulation (RY-element), circadian control (circadian) and root specific regulation (motif I) were predicted in the promoter regions of DlCDPs. Each promoter of DlCDPs had at least two elements related to growth and development. Among them, zein metabolism had the highest total number of O2-site elements among the promoters of DlCDPs, with 29 promoters containing them. Meristem expression was similar, with 38 O2-site elements in 25 promoters of DlCDPs. Interestingly, we found that seed specific regulation related RY-element was present in seven promoters of DlCDPs, suggesting that these DlCDPs could be potential genes involved in germination process in *D. latiflorus*.

### 3.6 TFBS in DlCDP promoters

**Figure 6:**
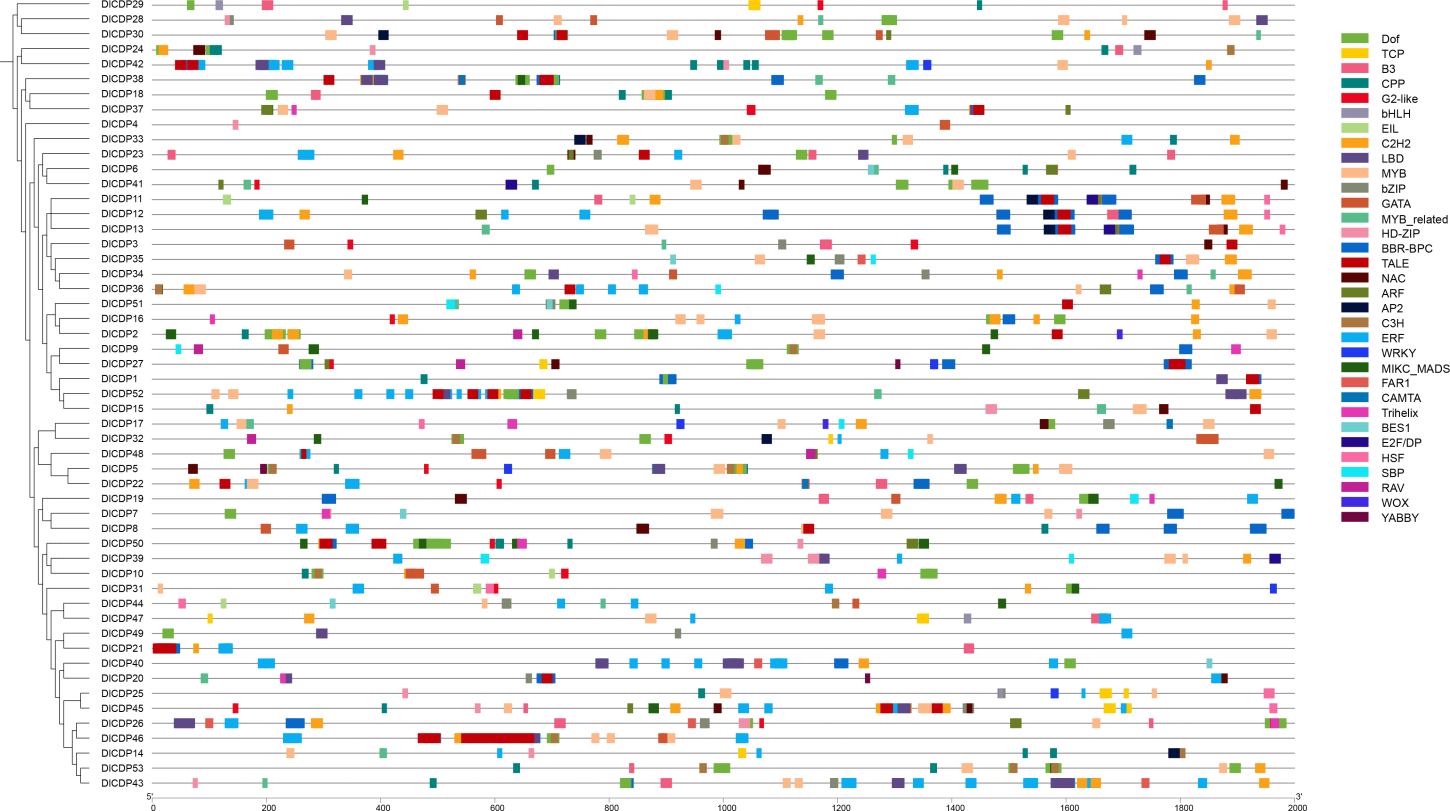
TFBS in the promoters of DlCDP genes.

We identified binding sites for 33 transcription factor (TF) families in the promoters of DlCDP family members (**Figure 6**). ERF binding sites were the most prevalent with 565, followed by BBR-BPC and Dof with 262 and 179 binding sites each, while WOX had only 2 binding sites, the least among them. Moreover, TFBS in each promoter of DlCDPs differed greatly in their types, numbers, and positions. There are 159 binding sites of TFs in *DlCDP43* promoter, while the *DlCDP4* promoter only contains 2 TFBS, which are GATA and HD-ZIP respectively. In previous studies, C2H2 TFs were shown to play an important role in bamboo shoot development (Guo et al., 2022), and TCP TFs were implicated in rhizome bud growth and shoot development (Jin et al., 2022). This led us to examine TFBS of these families in DlCDP promoters. Our analysis revealed 99 binding sites for C2H2 TFs across 38 promoters and 42 binding sites for TCP TFs across 8 promoters of DlCDPs.

### 3.7 Protein-protein interaction network of the DlCDP members

Based on the homologs of DlCDPs in *A. thaliana*, we constructed a protein-protein interaction (PPI) network among DlCDPs (**Figure S1**). The results revealed that DlCDP members interacted with each other. Cruciferins (CRUs) are seed storage proteins in *Arabidopsis* seedlings and have been implicated in response to oxidative stress (Nguyen et al., 2015). Some studies have shown that CRU3 is an ABA-responsive gene and is involved in the response to abiotic stress (Califar et al., 2020; Paponov et al., 2021). *DlCDP14*, *43* were identified as homologs of CRU3. Moreover, CRA1, CRU precursor, the homolog of DlCDP*26*,*46* and *53*, was reported to be involved in plant structure, growth, and development (Yu et al., 2010; Gan et al., 2019). CRU3 and CRA1 showed correlation in our network.

### 3.8 Transcriptome of DlCDP genes

#### 3.8.1 Expression patterns of DlCDPs in shoot bud and shoot

**Figure 7:**
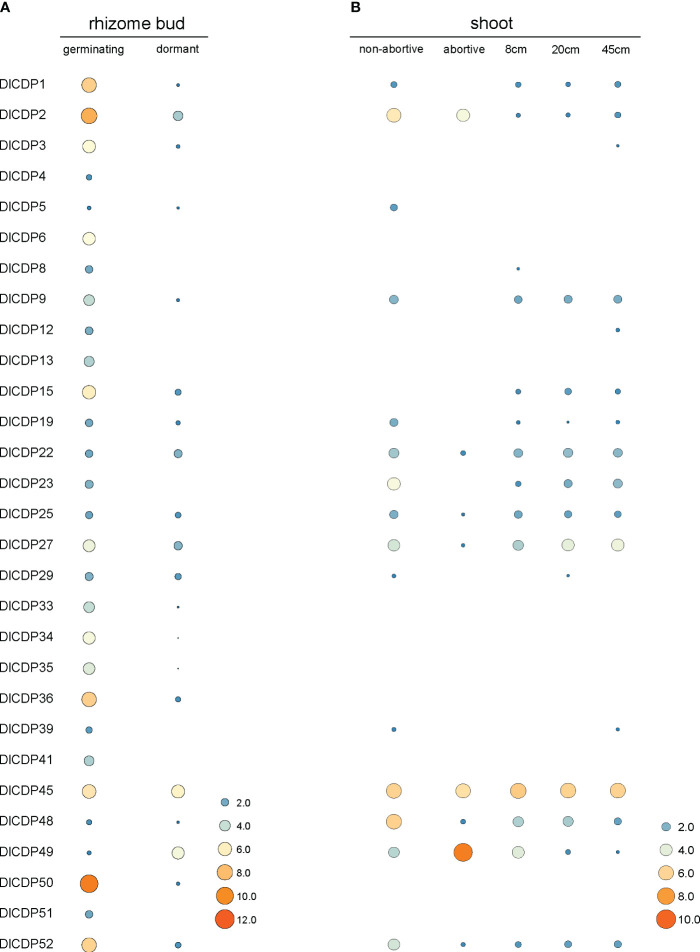
Expression patterns of DlCDP genes in rhizome buds **(A)**, and shoots **(B)**. The heatmap was drawn using TBtools software based on the log_2_(TPM + 1) values. The size and color scale represents expression levels from low (small; blue) to high (high; orange). TPM: transcripts per kilobase of exon model per million mapped reads.

**Figure 8:**
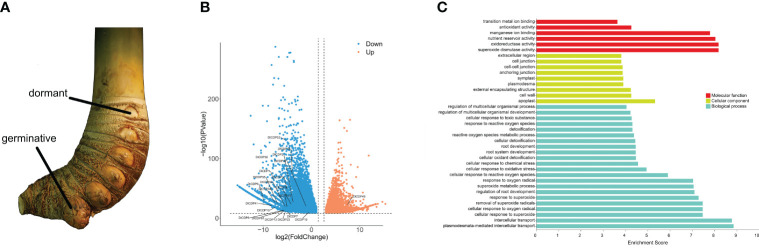
Rhizome buds used for transcriptome sequencing and differentially expressed genes (DEGs) among two organs. **(A)** *D*. *latiflorus* rhizome buds. **(B)** DEGs among dormant and germinative buds and DlCDPs among DEGs. **(C)** GO annotation of DlCDPs in DEGs.

To elucidate the roles of DlCDPs in bud growth and development, we measured their expression levels in germinating and dormant shoot buds (**Figures 7A**, **8A**). We identified 23 CDP family members as DEGs (**Figure 8B**). Among these, 22 genes showed down-regulation, while only one gene (*DlCDP49*) exhibited up-regulation. Notably, all down-regulated DEGs, except *DlCDP50* from subfamily V, belonged to subfamilies II and IV. Interestingly, the only up-regulated gene, *DlCDP49*, belonged to subfamily VII b, implying that bicupin and monocupin might have different biological functions. This indicates that CDP family members primarily function during the early stages of bud germination and development, with some potential involvement in bud dormancy.

To gain insights into the functions of DlCDPs, we performed GO annotation for the DEGs (**Figure 8C**). This analysis revealed their significant involvement in various biological processes, including oxygen metabolism, plant growth, and development. Specifically, these processes included responses to superoxide, cellular oxidant detoxification, root development, and cellular detoxification, among others. In terms of their cellular location, these genes were primarily found in the apoplast, cell wall, external encapsulating structures, plasmodesmata, and symplast, indicating their roles in structural components and communication within the plant. Regarding molecular function, these genes exhibited activities such as oxidoreductase activity, nutrient reservoir function, and manganese ion binding, suggesting their participation in vital biochemical and metabolic processes.

Since the degeneration rate of bamboo shoots is also a key factor affecting bamboo shoot yield, we further measured the expression levels of different DlCDPs between buds of different heights, abortive shoots and non-abortive shoots (**Figure 7B**). The results demonstrated that three CDP family members (*DlCDP23, 27, 48*) had significantly reduced expression levels in abortive shoots. In addition, a special DlCDP member, *DlCDP49*, showed relatively high expression levels in abortive shoots compared to non-abortive shoots.

#### 3.8.2 Validation of DlCDPs expression

**Figure 9:**
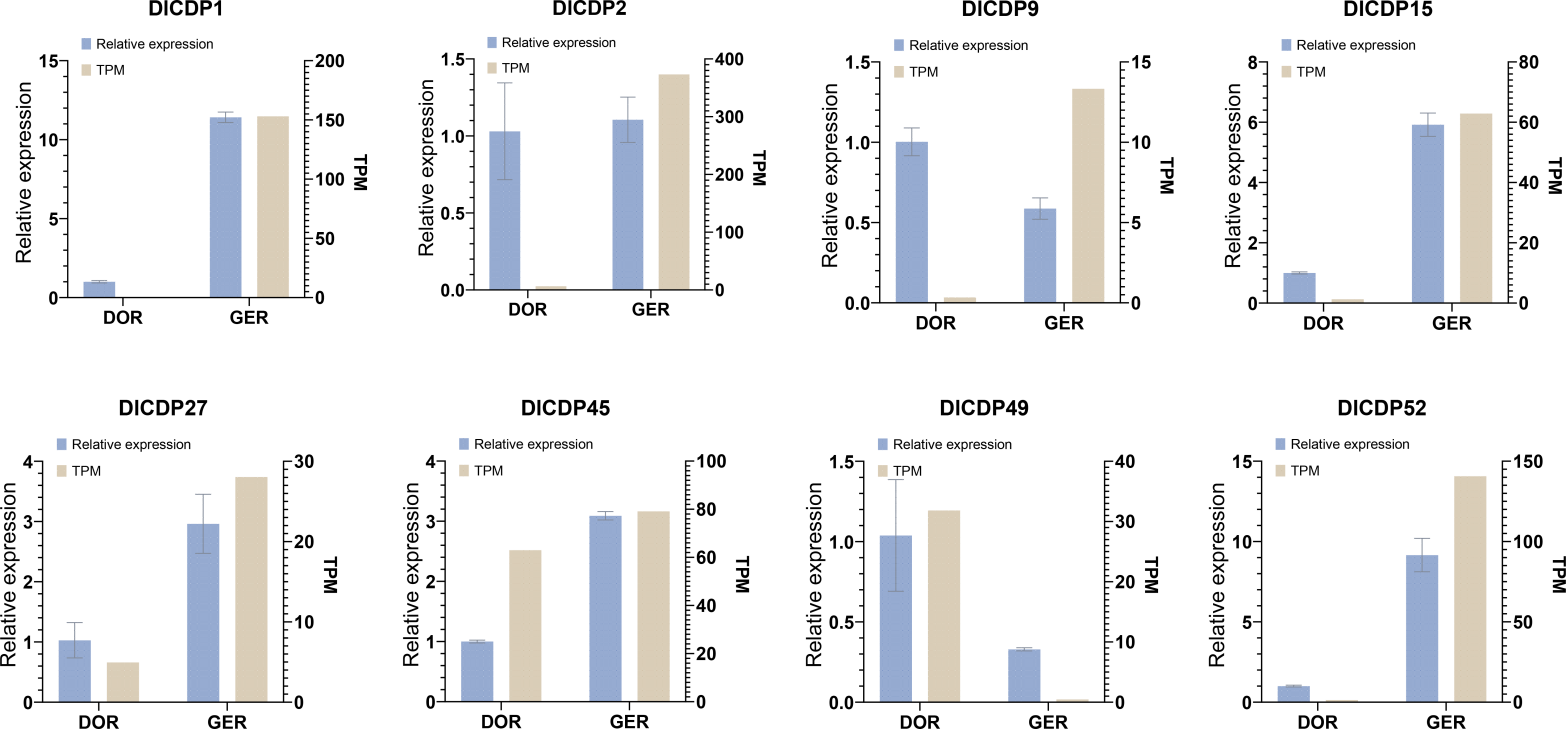
Relative expression patterns of eight selected DlCDP genes in rhizome buds. DOR, dormant rhizome buds; GER, germinative rhizome buds.

We further confirmed the relative expression of eight DlCDPs in the dormant and germinating bud groups by q-PCR. The results were consistent with the transcriptome data and showed that *DlCDP1*, *DlCDP15*, *DlCDP27*, *DlCDP45* and *DlCDP52* had the lowest expression levels in the dormant stage and significantly higher expression levels in the germinating stage (**Figure 9**), with 11.39, 5.92, 2.88, 3.09 and 9.15fold changes, respectively. In contrast, *DlCDP49* had significantly lower expression in the germinating stage than in the dormant stage, with a 0.32 fold change. In addition, *DlCDP2* had higher expression in the germination stage than in the dormancy stage, but q-PCR results showed that it had a small difference between the two stages, indicating that it played a role in both developmental stages. For *DlCDP9*, its q-PCR results showed a change pattern opposite to our transcriptome data, but most of our q-PCR results were consistent with the transcriptome data, indicating that our transcriptome data were reliable.

#### 3.8.3 Co-expression network of DlCDP gene

**Figure 10:**
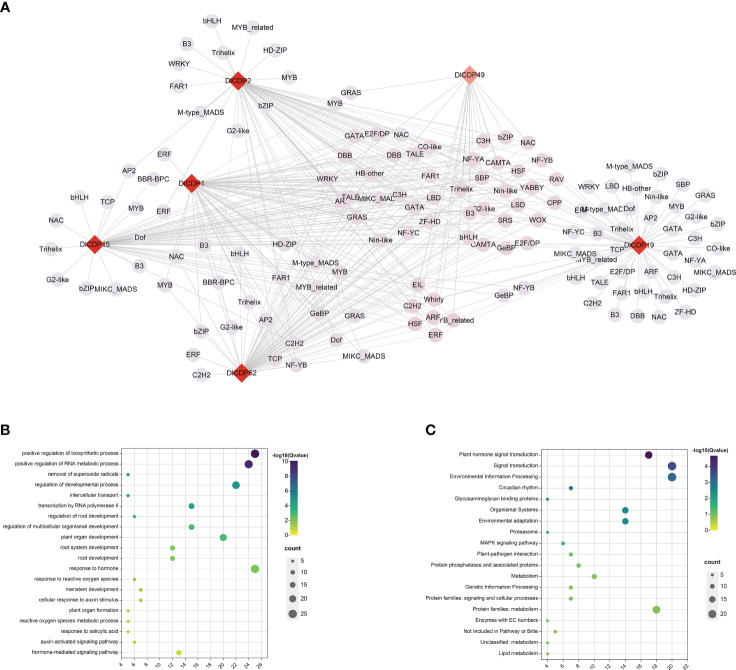
**(A)** DlCDP gene co-expression network constructed based on transcriptome data of *D. latiflorus* rhizome buds and shoots. Shades of color indicate the number of associated genes, with red indicating a high number of associated genes and pink indicating a low number of associated genes. Different shapes represent different types of genes, diamond represents six DlCDPs, and circles represent TFs. Solid line edges represent positive correlations and dotted line edges represent negative correlations. **(B)** GO enrichment of CDPs and TFs. **(C)** KEGG enrichment of CDPs and TFs.

Transcriptional regulation underlies many of the growth and developmental processes that shape plants as well as their adaptation to their environment (Strader et al., 2022). To explore the transcriptional regulatory network of DlCDPs in bamboo shoot development, we identified TFs in the shoot buds and shoot transcriptomes at different heights. A total of 56 transcription factor families containing 2489 sequences were identified. The most abundant gene families were bHLH, MYB and NAC, containing 218, 183 and 172 sequences respectively. We used Python scripts to analyze the expression levels of all transcription factors in the shoot buds and shoot transcriptome data to explore TFs co-expressed with DlCDP genes. A total of 148 TFs from different families were co-expressed with six DlCDP genes, with the most abundant transcription factor family being MYB (7), followed by B3, bHLH, bZIP, Trihelix, which had six each (**Figure 10A**, **Table S6**). DlCDPs may interact with these TFs to form a regulatory network and jointly participate in bamboo shoot development.

To verify the biological functions of DlCDP genes and other TFs in their co-expression network in bud growth and development, we performed GO and KEGG enrichment analysis for six DlCDP genes and 148 TFs in the co-expression network. The results showed that these genes were enriched in GO terms such as removal of superoxide radicals (5), regulation of developmental process (22), root development (12) and response to hormone (25) etc (**Figure 10B**). KEGG enrichment analysis results showed that these genes were enriched in KEGG pathways such as Plant hormone signal transduction (18), Environmental Information Processing (21), Glycosaminoglycan binding proteins (5), MAPK signaling pathway (7) and Lipid metabolism (5) etc (**Figure 10C**). These results indicate that the growth process of bamboo shoots has complex molecular biological effects, and the regulatory network of DlCDP genes plays an important role in plant growth and development.

## 4 Discussion

Rhizome sprouting empowers plants to regenerate aboveground parts from rhizomes, bolstering survival, spreading, and competitive advantage (Groff and Kaplan, 1988; Calvo et al., 2006; Shou et al., 2020). Bamboo is a typical RS species, whose shoot development involves complex molecular mechanisms (Zheng et al., 2022). CDP family includes GLP members that contain a single cupin_1 domain, which are related to hormone-mediated development processes (Murphy and Peer, 2022). GLPs have been identified in multiple species (Davidson et al., 2010; Cheng et al., 2014; Li et al., 2016; Zhang et al., 2022; Li et al., 2023), and whole CDP family members have also been identified in *B. napus* (He et al., 2022). In our cognition, the distribution and functional diversity of CDP family in ma bamboo have not been studied. In this study, 53 CDP genes were identified from ma bamboo (**Table S1**), and it was found that DlCDP is a diversified and complex gene family, with each subfamily having highly consistent gene structure and conserved motifs (**Figure 2**). Among them, the bicupins in DlCDP family all belong to the branch VII b, and these members have longer exon lengths (**Figure 2**). This may be due to the number of cupin_1 domains greatly affects the exon length and motif type of DlCDPs, which was also observed in rapeseed (He et al., 2022). Accordingly, the expression pattern of DlCDPs showed that bicupin member *DlCDP49* had a low expression level in well-developed tissues (**Figure 7**). This contrasted with the expression of monocupin members, indicating that it might play an important role in shoot bud dormancy and abortion. Therefore, we speculated that most DlCDP members promoted shoot bud germination and growth development, but some members might have opposite effects. Previous studies have shown that cupin_1 domain can bind to metal ions or other ligands (Khuri et al., 2001), so the number of cupin_1 domains may enable DlCDPs to exert multiple functions. However, DlCDPs have fewer bicupin members, and most of them have low expression levels. Specifically, the relationship between the number of cupin_1 domains and their gene function remains to be further elucidated.

During evolution, plants undergo gene duplication events, such as whole-genome duplication and tandem duplication, which often promote the expansion of gene families(Maere et al., 2005; Doyle et al., 2008), resulting in the diversity of gene function (Bowers et al., 2003). Chromosome localization showed that CDP genes were distributed in all subgenomes with equal numbers of chromosomes. Gene collinearity analysis revealed that there were many segmental duplications among subgenomes (**Figure 4**). Previous study showed that ma bamboo experienced three rounds of whole-genome duplication, which was considered to be the main driving force for the expansion of genes during the evolution of ma bamboo (Zheng et al., 2022). However, we found that subgenome C had the most DlCDP genes, and many members were distributed densely (**Figure 3**). Although these genes were not identified as tandem duplicated genes, some studies suggested that factors such as incomplete gene duplication make duplicated genes not equivalent redundant copies (Zhang et al., 2021). Therefore, we speculated that these DlCDPs were potential polymorphic tandem duplicated genes.

The number of orthologs of DlCDPs among species reflects the phylogenetic relationship among them. The species within the Poaceae share more conserved evolutionary relationships, and moso bamboo and ma bamboo, which are both members of the Bambusoideae, have the closest phylogenetic affinity (Tian et al., 2019). Most DlCDP members clustered with CDPs from *M. sinensis* and *O. sativa* in dense branches, while they had a relatively distant genetic relationship with *A. thaliana* (**Figure 1**). In addition, a larger proportion of DlCDPs in subgenome B formed orthologous pairs with CDPs from other species than those in subgenomes A and C (**Table 1**). This suggests that DlCDPs in subgenome B are more conserved, while DlCDPs in subgenome C underwent expansion after the divergence of ma bamboo from ancestors. Previous studies have shown that hexaploid woody bamboos have complex origins and experienced complex reticulate evolution(Guo et al., 2019). We conclude that this may account for the significant variation in the number and homology of DlCDPs among subgenomes and other species.

CDPs are essential for plant growth and development, especially in vegetative growth (Dunwell et al., 2004; Brunetti et al., 2022). Moreover, gene expression patterns are important indicators of gene functions. We quantified the expression patterns of DlCDPs in rhizome buds, shoots (**Figure 7**), and found that CDPs showed significant differences between dormant and germinative rhizome buds. This suggests that DlCDPs play important roles in shoot buds sprouting. In addition, TFs bind to specific DNA motifs to regulate the expression of target genes (Suter, 2020). Numerous TFs have been implicated in contributing to rhizome bud development (Shou et al., 2020). TF binding site prediction among promoter regions of DlCDP genes and co-expression network of DlCDPs with TFs indicates that the CDP family may be regulated by various TFs (**Figure 6**; **Figure 10A**). Among TFs, TCP and IDD TFs containing C2H2 domains have been reported to be involved in bamboo shoot growth(K. Jin et al., 2022; X. Guo et al., 2022), and our results suggested that CDPs interacted with them. This implies that these genes may play a significant role in bamboo shoot development. However, further verification and exploration are necessary to understand the interactions between CDPs and other TFs, as well as their regulatory mechanisms.

Previous studies have shown that CDPs have oxidative activities (Stipanuk et al., 2011). Specifically, GLPs, monocupin members of CDP, have multiple enzyme activities, such as oxalate oxidase (Sakamoto et al., 2015; Freitas et al., 2017), polyphenol oxidase(Cheng et al., 2014) and superoxide dismutase(Davidson et al., 2010). Function enrichment analysis of DlCDPs and co-expressed TFs unveiled roles in oxidative metabolism and hormone signaling (**Figures 10B**, **10C**), implying their involvement in ma bamboo shoot sprouting and growth. This suggests that DlCDPs might play important roles in ma bamboo shoot sprouting and growth by participating in oxidative metabolism. In addition, the PPI network showed that some DlCDP homologous proteins in *Arabidopsis* would synergistically respond to auxin regulation and affect rhizome growth and development (**Figure S1**).

Furthermore, there are numerous light-responsive elements within the DlCDP promoters (**Figure 5**), implying a potential connection between CDP activity and light conditions. The emergence of lateral rhizome buds enhances survival by providing advantages in nutrient and light acquisition in extreme environments (Calvo et al., 2006). Previous studies have indicated that some proteins with the cupin_1 domain possess sugar-binding capabilities (Khuri et al., 2001). Moreover, the transition from rhizome bud to bamboo shoot involves a gradual exposure to light, necessitating the involvement of genes with light-responsive capabilities. The expression pattern of DlCDPs significantly increases during rhizome bud germination (**Figure 7A**). A plausible speculation is that the sugar-binding or light-responsive processes mediated by CDPs influence the growth of rhizome buds. To conclude, CDP genes likely play a pivotal role in the rhizome bud germination process of Ma bamboo, though further experimental research is required to elucidate the precise physiological mechanisms.

## 5 Conclusion

In the context of rhizome sprouting, this study focused on exploring the significance of cupin domain-containing (CDP) genes within the ma bamboo genome. we identified and characterized 53 members of the cupin domain-containing (CDP) gene family from the ma bamboo genome. This family can be subdivided into seven subfamilies, which have consistent gene structures and motif distributions within each subcategory. Among these, we detected 12 pairs of DlCDP genes originating from segmental duplications, along with an additional 5 pairs resulting from tandem duplications embedded within the ma bamboo genome. Moreover, analysis of cis-acting elements and TFBS within the promoter regions of DlCDPs underscored an extensive interaction with a multitude of TFs. This interaction was substantiated by the co-expression network between DlCDPs and these TFs. As for functional implications, the enrichment analysis of DlCDPs and their co-expressed TFs suggested their intricate involvement in shaping the growth and development of rhizome buds and bamboo shoots, orchestrating their impact through diverse pathways. Furthermore, our observation of significant differential expression in numerous DlCDP genes between dormant and germinating rhizome buds strongly indicates their potential contributions to the process of rhizome bud germination in ma bamboo. Collectively, this study not only provides an understanding of CDPs within the ma bamboo but also yields valuable references for the intricate mechanisms governing rhizome bud germination and overall growth patterns in bamboo plants.

## Data availability statement

The datasets presented in this study can be found in online repositories. The names of the repository/repositories and accession number(s) can be found below: https://www.ncbi.nlm.nih.gov/, PRJNA988839 https://www.ncbi.nlm.nih.gov/, GSE155494 http://forestry.fafu.edu.cn/pub/Dla/index.html, transcriptome module.

## Author contributions

PZ: Conceptualization, Methodology, Formal Analysis, Writing – original draft. JY: Software, Writing – review & editing. DY: Investigation. YX: Data curation, Investigation. TH: Funding acquisition. JR: Project administration. YZ: Resources. LC: Supervision.

## Supplementary material

Supplementary Figure S1 Protein-protein interaction network for DlCDPs based on their orthologs in *A. thaliana*. DlCDP proteins were shown in brackets with *A. thaliana* orthologs.

Supplementary Table S1 Basic information for the *D. latiflorus* CDP gene family members.

Supplementary Table S2 The detailed information of conserved motifs in DlCDPs.

Supplementary Table S3 Subfamily and duplication type for gene pairs of DlCDPs.

Supplementary Table S4 Ka/Ks ratios for paralogous (Dl-Dl) and orthologous (Dl-Os, Dl-Ms, Dl-Pe) gene pairs.

Supplementary Table S5 q-PCR primers in *D. latiflorus*.

Supplementary Table S6 List of transcription factors co-expressed with DlCDP genes.
