# Supplementary figures and images for: Identification and characterization of the cupin_1 domain-containing proteins in ma bamboo (Dendrocalamus latiflorus) and their potential role in rhizome sprouting
Source: Front Plant Sci. 2023 Oct 16;14:1260856. doi: 10.3389/fpls.2023.1260856 (PMC10614299; doi:10.3389/fpls.2023.1260856)

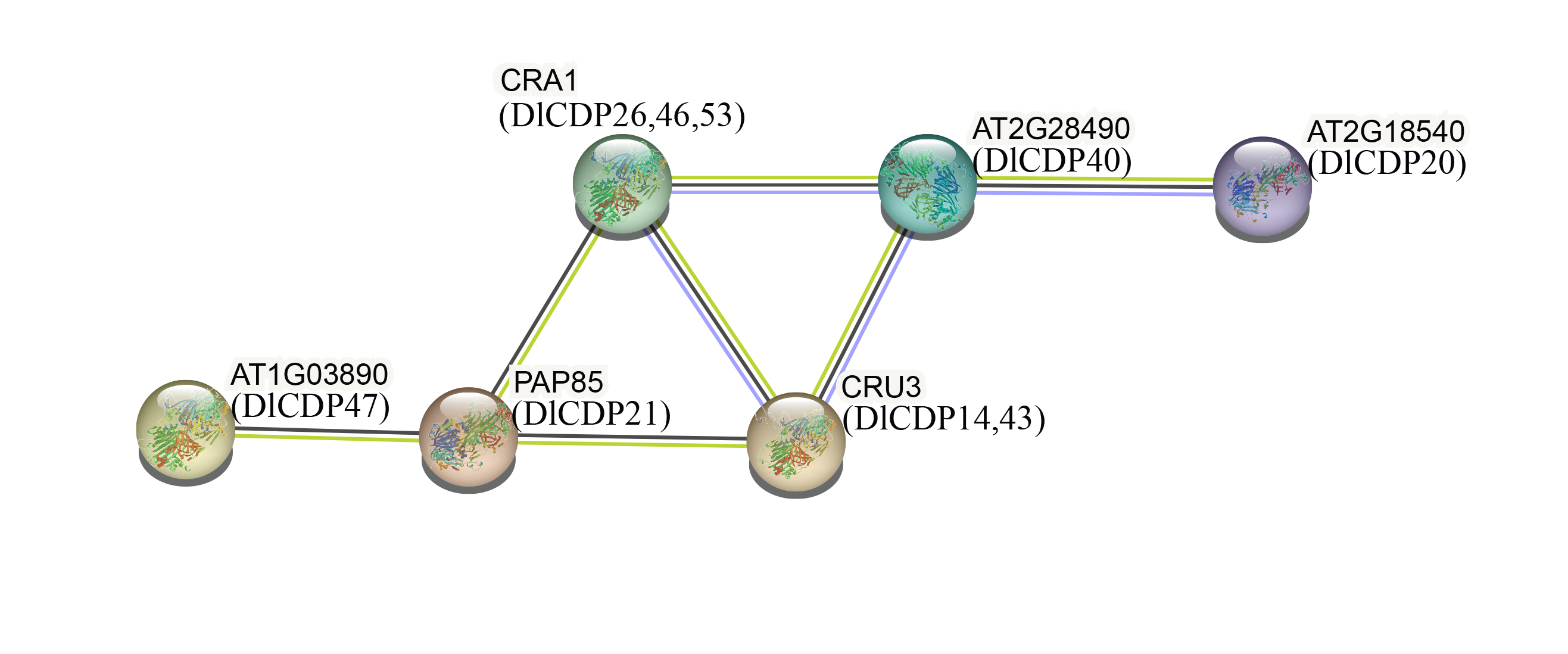

Supplement: Supplementary Figure S1 — Protein-protein interaction network for DlCDPs based on their orthologs in A. thaliana. DlCDP proteins were shown in brackets with A. thaliana orthologs. [file DataSheet_1.zip › Image 1 (50).TIF]
